# Serine/Glycine Lipid Recovery in Lipid Extracts From Healthy and Diseased Dental Samples: Relationship to Chronic Periodontitis

**DOI:** 10.3389/froh.2021.698481

**Published:** 2021-07-16

**Authors:** Frank C. Nichols, Kruttika Bhuse, Robert B. Clark, Anthony A. Provatas, Elena Carrington, Yu-Hsiung Wang, Qiang Zhu, Mary E. Davey, Floyd E. Dewhirst

**Affiliations:** ^1^Department of Oral Health and Diagnostic Sciences, University of Connecticut School of Dental Medicine, Farmington, CT, United States; ^2^Departments of Immunology and Medicine, University of Connecticut School of Medicine, Farmington, CT, United States; ^3^Center for Environmental Sciences and Engineering, University of Connecticut, Storrs, CT, United States; ^4^Department of Craniofacial Sciences, University of Connecticut School of Dental Medicine, Farmington, CT, United States; ^5^Department of Oral Biology, University of Florida College of Dentistry, Gainesville, FL, United States; ^6^Department of Microbiology, The Forsyth Institute, Cambridge, MA, United States; ^7^Department of Oral Medicine, Harvard School of Dental Medicine, Infection and Immunity, Boston, MA, United States

**Keywords:** *Porphyromona gingivalis*, bacterial serine/glycine lipids, chronic periodontitis, dental tissues, Lipid 1256

## Abstract

Toll-like receptor 2 (TLR2) activation has been implicated in the pathogenesis of periodontal disease but the identity of the TLR2 agonists has been an evolving story. The serine/glycine lipids produced by *Porphyromonas gingivalis* are reported to engage human TLR2 and will promote the production of potent pro-inflammatory cytokines. This investigation compared the recovery of serine/glycine lipids in periodontal organisms, teeth, subgingival calculus, subgingival plaque, and gingival tissues, either from healthy sites or periodontally diseased sites. Lipids were extracted using the phospholipid extraction procedure of Bligh and Dyer and were analyzed using liquid chromatography/mass spectrometry for all serine/glycine lipid classes identified to date in *P. gingivalis*. Two serine/glycine lipid classes, Lipid 567 and Lipid 1256, were the dominant serine/glycine lipids recovered from oral Bacteroidetes bacteria and from subgingival calculus samples or diseased teeth. Lipid 1256 was the most abundant serine/glycine lipid class in lipid extracts from *P. gingivalis, Tannerella forsythia*, and *Prevotella intermedia* whereas Lipid 567 was the most abundant serine/glycine lipid class recovered in *Capnocytophaga* species and *Porphyromonas endodontalis*. Serine/glycine lipids were not detected in lipid extracts from *Treponema denticola, Aggregatibacter actinomycetemcomitans*, or *Fusobacterium nucleatum*. Lipid 1256 was detected more frequently and at a significantly higher mean level in periodontitis tissue samples compared with healthy/gingivitis tissue samples. By contrast, Lipid 567 levels were essentially identical. This report shows that members of the Bacteroidetes phylum common to periodontal disease sites produce Lipid 567 and Lipid 1256, and these lipids are prevalent in lipid extracts from subgingival calculus and from periodontally diseased teeth and diseased gingival tissues.

## Introduction

Periodontitis afflicts the majority of individuals by the age of 50 and although the microbial etiology has been extensively characterized, the underlying mechanisms by which microbes promote tissue destructive processes are only partially understood. The complexity of the subgingival microbial flora and the multitude of host responses in diseased periodontal tissues has generated many potential mechanisms to explain tissue destruction in periodontitis. Our laboratory has focused on the relationship between novel lipids of *Porphyromonas gingivalis* and their capacity to engage the innate immune system as a mechanism for promoting inflammation and tissue destruction in periodontitis. These studies were initiated in part because a prior report showed a doubling of complex lipids containing a unique branched chain bacterial fatty acid (3-OH *iso* C_17:0_) in subgingival plaque samples from periodontitis sites when compared with gingivitis sites [1]. However, LPS which also contains 3-OH *iso* C_17:0_, was found to be two-fold lower in the same plaque samples from periodontitis sites compared with gingivitis sites [1]. Because this unique bacterial fatty acid is recovered essentially only in complex lipids and LPS of bacteria of the Bacteroidetes phylum [1–5], the preferential accumulation of complex lipids containing 3-OH *iso* C_17:0_ in subgingival plaque of periodonitis sites suggested a lipid dysbiosis and led to the characterization of lipid classes of *P. gingivalis* that contain this unique branched fatty acid. The first lipids to be characterized were the phosphorylated dihydroceramides [6] but more recently, serine/glycine lipids of *P. gingivalis* were characterized because these lipids were observed to engage the innate immune receptor, Toll-like receptor 2 (TLR2) [2–4]. It should be emphasized that *P. gingivalis* is reported to mediate bone loss in experimental animals through engagement of TLR2 [7–9]. The purpose of the present study was to evaluate bacterial and dental samples for the recovery of the novel serine/glycine lipids of *P. gingivalis*.

**Figure 1:**
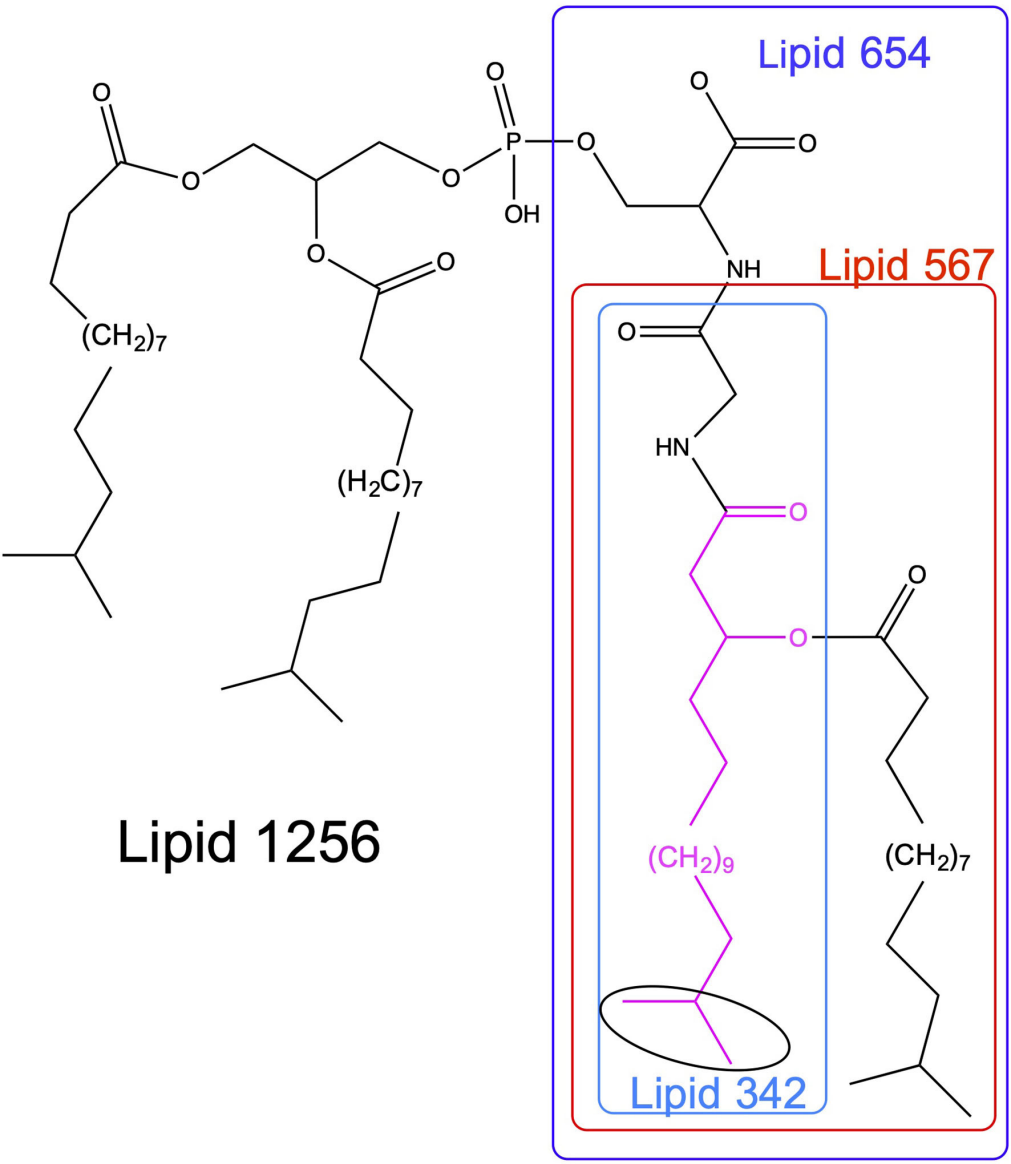
Structures of serine/glycine lipids produced by *P. gingivalis*. The structures of the serine/glycine lipids are depicted for the dominant lipid species within each lipid class. Lipid 430 contains a serine attached to the glycine of Lipid 342 and is not included in this figure. All of the serine/glycine lipid classes contain a Lipid 342 core structure, therefore they appear to be sequentially synthesized in Bacteroidetes organisms. Highlighted in pink is 3-hydroxy *iso*branched C_17:0_ (3-OH *iso* C_17:0_) and the *iso*branched portion of the aliphatic chain is encircled in black.

The serine/glycine lipids [2, 3] of *P. gingivalis* are amenable to identification and quantification using current mass spectrometric methods. Each lipid class is named based on the mass of its dominant species. Two serine-glycine dipeptide lipids [2] and two glycine lipids [3] (Figure 1) have been characterized from *P. gingivalis*. Each of these lipid classes contains a core structure (Lipid 342) consisting of glycine amide-linked to 3-OH *iso* C_17:0_ (shown in pink, Figure 1). Importantly, each lipid class is comprised of at least three species due to subsitution of branched or straight chain fatty acids of differing lengths, but the dominant lipid species in each class are shown in Figure 1. The most recently characterized serine lipid class, called Lipid 1256 [4], is the primary focus of this study. As shown in Figure 1, this lipid is composed of Lipid 654 supplemented with a diacylated phosphoglycerol held in phosphoester linkage to the serine of Lipid 654.

Like LPS, an important feature of the serine/glycine lipids is they elicit marked biological responses in host cells. Although a survey of cellular effects is incomplete, recent work has shown that specific serine/glycine lipids can alter osteoblast [10] and dendritic cell function [2], and perhaps affect other inflammatory and connective tissue cells. The Lipid 1256 class was recently shown to activate cytokine secretory responses in human peripheral blood monocytes [4]. Most importantly, these lipids engage human TLR2 [2] as well as its heterodimeric receptor TLR2/6 [3, 4]. Among the serine/glycine lipids, Lipid 1256 is the most potent ligand for TLR2 and TLR2/6 [4]. These lipid classes can be produced by essentially all members of the Bacteroidetes phylum, including oral Bacteroidetes [4]. In order to show disease relevance for these lipids, the present investigation surveyed the recovery of these lipids in common subgingival organisms, including those associated with periodontal disease and dental samples from either periodontally healthy or periodontitis disease sites.

## Materials and Methods

### Preparation of Bacteria

*Porphyromonas gingivalis* (ATCC #33277, type strain, W83, and 381), *Prevotella intermedia* (VPI 8944, generous gift of Dr. L.V. Moore, VPI), and *Porphyromonas endodontalis* (ATCC #35406, type strain) were grown in basal [peptone, trypticase and yeast extract, (BBL)] medium supplemented with hemin and menadione (Sigma-Aldrich, St Louis) and brain heart infusion as previously described [4, 5]. *Aggregatibacter actinomycetemcomintans* and *Tannerella forsythia* were generously provided by Dr. Sigmund Socransky, Forsyth Institute (deceased). *Capnocytophaga* species were a generous gift of Dr. Paulette Tempro. *Treponema denticola* (ATCC #35405) was grown in ATCC 1494 medium. *Fusobacterium nucleatum* (ATCC 25586, VPI 4355) was grown in Trypticase Soy Broth (BBL). Bacteria were harvested by centrifugation (3,000 × g × 20 min) and lyophilized. At the time of lipid extraction, samples of bacterial pellets were thawed and extracted using the phospholipid extraction procedure described below.

### Collection of Extracted Teeth, Subgingival Plaque Samples, and Gingival Tissue Samples

Informed consent was obtained from patients in accordance with Institutional Review Board (IRB) policy of the University of Connecticut Health Center (UCHC) before collection of gingival tissue samples and subgingival plaque samples. Gingival tissue samples were of two categories: crown lengthening procedures at sites with no attachment loss and either health or gingivitis, or gingival tissue samples recovered from sites with severe **periodontitis (stage III or IV)** demonstrating pocketing of >5mm and >50% bone loss, and moderate to severe gingival inflammation. De-identified discarded extracted teeth were obtained and were used in accordance with UCHC IRB policy, and following lipid extraction, the teeth were autoclaved and discarded. Teeth with severe attachment loss due to periodontitis demonstrated adherent and usually grossly apparent subgingival calculus. Impacted third molars removed in the Department of Oral and Maxillofacial Surgery were obtained and were used in accordance with UCHC IRB policy for this study. Gross adherent soft tissue was removed from calculus-contaminated teeth before lipid extraction but some adherent soft tissue remained attached to the teeth. Subgingival plaque samples were obtained by first removing supragingival plaque followed by placement of coarse endodontic paper points into interproximal sulcuses demonstrating severe attachment loss. Subgingival plaque samples were not collected from healthy/gingivitis sites due to the low amount of lipid recovered for pooled samples from individual subjects. Subgingival plaque contents were collected for ~10 s from interproximal pockets. Paper points containing subgingival plaque were pooled for each individual (two subjects) with **generalized stage III to IV periodontitis** and represented sampling of at least two dozen posterior interproximal sulcuses. Gingival tissue samples were surgically excised from either crown lengthing sites or severe periodontitis sites and were immediately frozen.

### Lipid Extraction Procedure

Lipids were extracted from bacterial pellets, teeth, subgingival plaque, and homogenized gingival tissue samples using the phospholipid extraction procedure of Bligh and Dyer [11] as modified by Garbus [12]. Specifically, 0.5 ml of H_2_0 + 2 ml of MeOH:CHCl_3_ (2:1 v/v) was added to each bacterial sample, subgingival plaque or calculus sample, and the samples were vortexed. Gingival tissue samples were thawed and homogenized in this solvent phase of the Bligh and Dyer extraction procedure. The remaining phospholipid extraction procedure was completed once the gingival tissue samples were thoroughly homogenized as previously described [13]. For individual teeth, the extraction volumes were doubled to ensure that the entire tooth surface was exposed to extraction solvent. After 12 h, 0.75 ml of 2 N KCl + 0.5 M K_2_HPO_4_ and 0.75 ml CHCl_3_, or double these volumes for teeth, were added and the samples were vortexed. After centrifugation (2,500 × g × 10 min), the lower organic phase was carefully removed and dried under nitrogen.

### LC-MS Method

**Figure 2:**
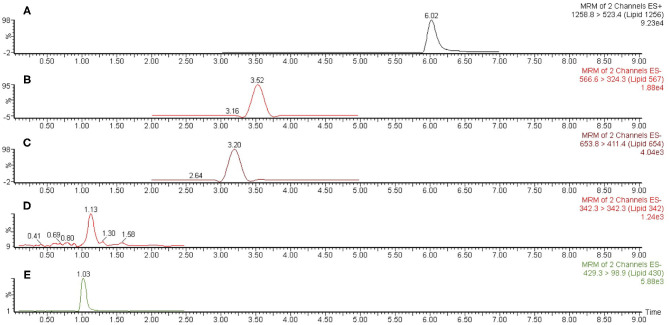
Representative liquid chromatography-mass spectrometric (LC-MS) scan of *P. gingivalis* (ATCC 33277) total lipids. A sample of *P. gingivalis* total lipids was dissolved in methanol and analyzed by LC-MS as described in section Materials and Methods. Scan **(A)** represents Lipid 1256 monitored as the *m/z* 1258.8 to 523.4 positive ion multiple reaction monitoring (MRM) transition, scan **(B)** represents Lipid 567 monitored as the *m/z* 566.6 to 324.3 negative ion MRM transition, scan **(C)** represents Lipid 654 monitored as the *m/z* 653.8 to 411.4 negative ion MRM transition, scan **(D)** represents Lipid 342 monitored as the *m/z* 342.3 negative ion scan and scan E represents Lipid 430 monitored as the *m/z* 429.3 to 98.9 negative ion MRM transition. The MRM ion peaks were electronically integrated in order to calculate the relative abundance of each serine/glycine lipid class.

*Liquid chromatography-mass spectrometry (LC-MS) analysis*—Dried lipid samples were dissolved in a small volume of methanol and vortexed. The samples were centrifuged and transferred to autosampler tubes. Analysis of lipids utilized mass spectrometric instrumentation located in the Center for Environmental Sciences and Engineering at the University of Connecticut, Storrs as previously described [4]. Routine analysis of lipid samples used a Waters Acquity™ UPLC® coupled with an Acquity™ TQD™ tandem mass spectrometer (Waters Co., Milford, MA). An Acquity™ UPLC CSH C18 (1.7 μm, 2.1 × 100 mm) column, heated to 50°C and with a sample injection volume of 20 μL on a 20 μL loop, was utilized for analyte separation. The mobile phase consisted of 10 mM ammonium formate, 0.1% formic acid in 40% water/60% acetonitrile (solvent A) and 10 mM ammonium formate, 0.1% formic acid in 90% isopropyl alcohol/10% acetonitrile (solvent B) was employed for gradient elution. Initial flow of 75% solvent A was held for 3.0 min before increasing linearly to 100% solvent B until 8.0 min, after which the column was reconditioned to the initial state for another 1.0 min. Total run time was 9.0 min with a constant flow rate of 0.4 ml/min. A representative LC-MS scan of *P. gingivalis* total lipids is depicted in Figure 2.

The detection and quantification of serine/glycine lipids was performed in negative ESI-MS/MS mode (MRM) for all serine glycine lipids except for Lipid 1256 that was detected in positive ESI-MS/MS mode. Ions were quantified using the Waters, Inc. IntelliStart™ software for analyte signal optimization. Statistical analysis for obtaining calibration and quantification results for all compounds was performed using Waters QuanLynx™, which was included in the MassLynx™ software v.4.2. Each serine/glycine lipid class in dental or microbial samples was identified based on retention time of purified or synthetic standards of each lipid class. Each lipid class was further verified by quantifying the characteristic molecular parent ion for each lipid class as well as by quantifying the dominant MS/MS ion transition characteristic for each serine/glycine lipid class. Ion abundances for each serine/glycine lipid parent ions or ion transitions measured by multiple reaction monitoring, were electronically integrated using MassLynx™ software. Parameters for the mass spectrometer were set as follows: capillary voltage, 2.0 kV; cone voltage, 30 V; desolvation temperature, 400°C; source temperature, 120°C; desolvation gas flow, 750 L/h; collision gas flow, 0.2 ml/min.

### Statistical Methods

The results depicted in **Figure 4** were analyzed by one factor ANOVA using prism. The results for the diseased samples were normally distributed but for the control samples, most sample categories were not normally distributed. For **Figures 4A,B**, the results are treated as normally distributed and the means for sample groups are depicted as histogram bars with standard deviations. One factor ANOVA was performed and pairwise comparisons were evaluated using Tukey's multiple comparisons. The results were also analyzed by Kruskal-Wallis one factor ANOVA with pairwise comparisons using Dunn's test and the significant pairwise comparisons were the same for both the Dunn's and Tukey's multiple comparisons. The number of samples analyzed are included in sections Results and Figure Legends.

## Results

**Figure 3:**
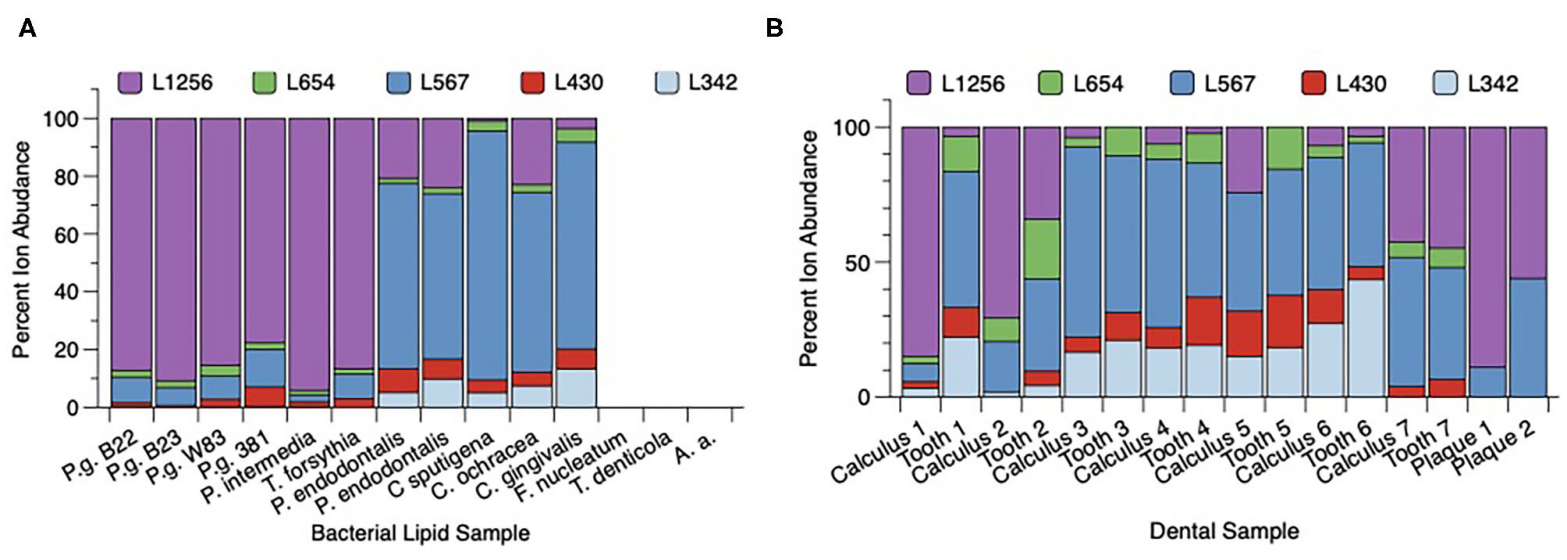
Distribution of serine/glycine lipids in subgingival organisms and dental samples. **(A)** Bacteria were grown in broth culture and processed as described in section Materials and Methods. Individual bacterial lipid extracts were analyzed by LC-MS as described in section Materials and Methods. Recovery of individual lipid classes is depicted as a percent of the total serine/glycine lipids recovered in each bacterial lipid sample. Serine/glycine lipids were recovered in *Porphyromonas gingivalis* (P.g.), *Prevotella* (P.) *intermedia, Tannerella* (T.) *forsythia, Porphyromonas endodontalis*, and three *Capnocytophaga* (C.) species. Serine/glycine lipids were not detected in *Fusobacterium* (F.) *nucleatum, Treponema* (T.) *denticola* or *Aggregatibacter actinomycetemcomitans* (A.a.). **(B)** Recovery of serine/glycine lipids in lipid extracts from paired tooth/subgingival calculus samples, and lipid extracts from two pooled subgingival plaque samples isolated from two patients with generalized severe periodontitis. Seven hopeless teeth diagnosed with severe chronic periodontitis were extracted and the teeth were lyophilized. Dark subgingival calculus was removed from each tooth using sterile scalers and the paired tooth/subgingival calculus samples were individually extracted. Frame **B** compares the percent serine/glycine lipid recovery for each paired calculus and tooth sample. Except for tooth 1, the distribution of serine/glycine lipids is similar between subgingival calculus and tooth samples. Paper point samples were collected using sterile endodontic paper points inserted into interproximal pockets and were pooled for two individual patients demonstrating generalized severe chronic periodontitis. The paper point samples were then extracted for total lipids as described in section Materials and Methods. Lipid 567 and Lipid 1256 were the only serine/glycine lipids detected in lipid extracts from pooled subgingival plaque samples.

Total lipid extracts from *P. gingivalis* (two batches of 33277, W83, and 381) as well as *Prevotella intermedia, T. forsythia, Porphyromonas endodontalis, Capnocytophaga sputingena, C. ochracea*, and *C. gingivalis* were evaluated for relative levels of serine/glycine lipids (Figure 3A). Three patterns were observed for the serine/glycine lipid distributions in these organisms. Lipid 1256 was dominant in *P. gingivalis, Prevotella intermedia* and *T. forsythia*, Lipid 567 was dominant in *Capnocytophaga* species and *P. endodontalis* and serine/glycine lipids were not recovered in *Fusobacterium nucleatum, Treponema denticola*, and *Aggregatibacter actinomycetemcomitans*. Other serine/glycine lipids recovered from these bacteria were relatively minor lipid consituents.

Next, seven diseased teeth were extracted from patients and most subgingival calculus was removed with scalers. The individual teeth and calculus samples were then extracted for total lipids (Figure 3B). The recovery of serine/glycine lipids in the paired teeth/calculus samples showed that Lipid 1256 and Lipid 567 varied considerably between the tooth samples. However, for most tooth samples, the distribution of serine/glycine lipids was similar between calculus and teeth lipid extracts. Teeth #3-6 showed higher levels of Lipid 567 when compared with Lipid 1256. Tooth 1 showed a major shift in the distribution of serine/glycine lipids between the lipid extract recovered from the diseased tooth compared with the lipids recovered from removed calculus. Because these teeth were de-identified, it was not possible to determine how recently the teeth had received scaling and root planing prior to tooth extraction. Analysis of two pooled subgingival plaque samples collected from two independent donors with **generalized stage III to IV periodontitis** revealed essentially only Lipid 567 and Lipid 1257.

**Figure 4:**
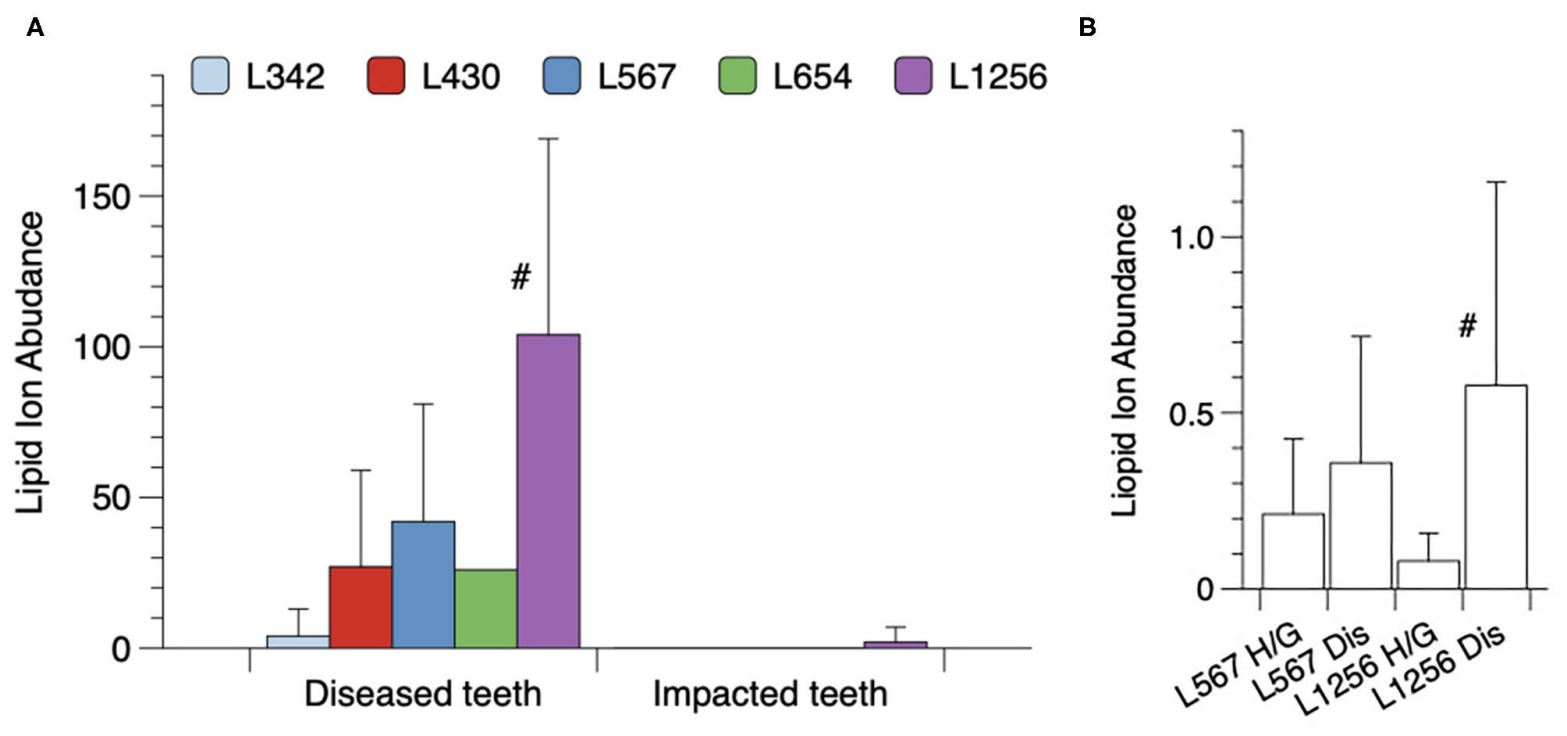
Recovery of serine/glycine lipids on extracted teeth or recovered in surgical samples of gingival tissues. **(A)** Categories of teeth included either fully impacted third molars or hopeless teeth extracted due to **stage III or IV periodontitis**. The periodontitis-afflicted teeth were laden with gross amounts of dark subgingival calculus. Lipid extracts from individual teeth were dissolved in 120 ul of methanol and the samples were analyzed by LC-MS as described. Results are depicted as relative serine/glycine lipid ion abundances for five individual diseased (Dis) teeth and five individual impacted third molars (Imp) with a significant difference noted only for Lipid 1256 (#, **by Tukey's multiple comparisons test**). **(B)** Excised gingival tissue samples were collected and analyzed as described in section Materials and Methods. Equivalent amounts of total lipid extract from each gingival tissue sample were analyzed by LC-MS. Histogram bars represent the mean ion abundances of each lipid class with standard deviations indicated by vertical error bars. A total of 7 healthy/gingivitis and 10 severe periodonitis samples were analyzed. The number of samples positive for Lipid 1256 was 8 out of 10 for diseased (Dis) samples and 2 out of 7 for the healthy/gingivitis (H/G) samples. The mean level of Lipid 1256 was significantly elevated in diseased samples compared with healthy/gingivitis samples (#, **Tukey's multiple comparisons test**). Serine/glycine lipid classes other than Lipid 1256 and Lipid 567 were not detected in lipid extracts from diseased gingival tissue samples.

Next, lipids were extracted from hopeless teeth that were heavily laden with subgingival calculus and these lipid extracts were compared with those from fully impacted teeth that were surgically extracted. Figure 4A shows the mean recoveries of each lipid class for the averaged results **from five periodontally diseased teeth** and five impacted teeth. Lipid 1256 was the dominant lipid class recovered in lipid extracts from the periodontally-diseased teeth. By contrast, lipid extracts from impacted teeth demonstrated negligible levels of the serine/glycine lipids. **Using one factor ANOVA with Tukey's multiple comparisons, only the Lipid 1256 lipid levels were significantly increased for the diseased teeth when compared with impacted teeth**. Because the bacterial lipids were contaminated with host-derived lipids from teeth, it was not possible to calculate the absolute amounts of the serine/glycine lipids recovered.

Finally, the levels of the serine/glycine lipids recovered in lipid extracts from gingival tissue samples excised from sites diagnosed with severe periodontitis or from healthy/gingivitis sites undergoing crown lengthening procedures, are shown in Figure 4B. For this evaluation, 300 μg of lipid from each tissue extract was dissolved in 120 ul of methanol and each sample was analyzed for the lipids of interest. Of the serine/glycine lipids analyzed, only Lipid 567 and Lipid 1256 were detected using the LC-MS method described in section Materials and Methods. Lipid 567 was detected with approximately the same frequency and the same mean level in tissue samples from healthy sites and periodontally diseased sites. However, Lipid 1256 was detected more frequently **(8 out of 10 samples in diseased tissue samples vs. 2 out of 7 in healthy/gingivitis samples)** and a signficantly higher mean level was observed in diseased gingival tissues when compared with healthy tissue samples. These results indicate that a selective deposition of Lipid 1256 may be occurring in diseased gingival tissues at periodontal disease sites.

## Discussion

The serine/glycine lipid classes Lipid 654 and Lipid 430 were originally reported as constituents of non-oral bacteria from the genera *Flavobacterium* and *Cytophaga* [14–18], but further studies have determined that these lipids are constituents of all members of the Bacteroidetes phylum evaluated to date. Lipid 342 and Lipid 567 were reported next [2, 3] and Lipid 1256 was the most recently described serine/glycine lipid class recovered from *P. gingivalis* [4]. Our limited survey of oral subgingival organisms indicates that representative members of the Bacteroidetes phylum of the oral cavity produce most or all of these serine/glycine lipids. Of particular importance is that both *P. gingivalis* and *T. forsythia* are members of the red complex organisms strongly associated with the expression of periodontitis [19], and both preferentially produce Lipid 1256 as the dominant serine/glycine lipid class. *Treponema denticola*, the third microbe of the red complex, does not produce any of the serine/glycine lipids. Although *Aggregatibacter actinomycetemcomitans* recovery is typically highest in subgingival plaque **from periodonititis sites with a molar incisor distribution**, it also does not produce these lipids. Analysis of these lipids in dental samples, particularly samples from periodontitis sites, revealed that Lipid 567 and Lipid 1256 predominate in lipid extracts from either teeth laden with gross amounts of subgingival calculus, calculus removed from roots of extracted teeth, subgingival plaque samples or gingival tissue samples. However, our results indicate that lipid extracts from periodontally diseased teeth and subgingival calculus contain either predominantly Lipid 567 or Lipid 1256, but typically not both. Lipid 567 was abundant and Lipid 1256 was minimal in lipid extracts from *Capnocytophaga* species and *P. endodontalis* but not in *P. gingivalis* and *T. forsythia*. The reverse was observed for Lipid 567 and Lipid 1256 recovery in *P. gingivalis* and *T. forsythia*. It is not known if Lipid 1256 can be converted to Lipid 567 in subgingival plaque. Regardless, additional work will be required to determine the microbial basis for the two distinct patterns of serine/glycine lipid accumulation observed in subgingival calculus vs. that observed on diseased teeeth.

Deposition of other *P. gingivalis* complex lipids, including phosphorylated dihydroceramides, in gingival tissues was demonstrated in two prior reports [13, 20]. Our results here show that the recently discovered Lipid 1256 is selectively and significantly elevated in diseased gingival tissue samples when compared with healthy/gingivitis samples. Importantly, while Lipid 1256 was observed at the lower end of the detection range with only two of seven lipid extracts from healthy/gingivitis tissue samples, Lipid 1256 was observed in eight of ten severe periodontitis gingival tissue samples and the mean level was significantly elevated over the healthy/gingivitis tissue lipid extracts. Therefore, the data indicate that Lipid 1256 is elevated in gingival tissue samples compared with healthy/gingivitis sites. Invasion of *P. gingivalis* into gingival tissues has been reported [21–26] and could account for elevated Lipid 1256 levels in diseased gingival tissues. However, the distribution of serine/glycine lipids in diseased gingival tissues is unlike that observed in the organisms themselves. Lipid 567 is relatively low in *P. gingivalis* and *T. forsythia* cells, yet the mean level of Lipid 567 is comparable to Lipid 1256 in the diseased gingival tissue samples. This could argue that it is not as simple as serine/glycine lipid accumulation due to either *P. gingivalis* or *T. forsythia* whole cell invasion into diseased tissues at periodontitis sites. Since bacterial membrane lipids are dynamic and altered in response to environmental parameters and there are a number of oral Bacteroidetes with the potential to produce these lipids, it is not yet clear why these particular lipids accumulate in diseased tissues. These results also suggest that Lipid 567 recovery in diseased gingival samples may not be predictive of periodontitis expression.

The significant increase in percent of 3-OH *iso* C_17:0_ in organic solvent extracts of periodontitis subgingival plaque samples reported previously [1] is a strong indicator of a lipid dysbiosis. Here we show that serine/gycine lipids, particularly Lipid 567 and Lipid 1256, are likely contributing to the elevated recovery of 3-OH *iso* C_17:0_-containing lipids previously reported in subgingival plaque at periodontitis sites compared with gingivitis sites [1]. The reason for the increased recovery of lipids containing this particular fatty acid is likely due in part to the increase in periodontopathic organisms *P. gingivalis, P. intermedia*, and *T. forsythia* producing high levels of phosphorylated dihydroceramide lipids along with Lipid 1256 as well as less disease-associated *P. endodontalis* and *Capnocytophaga* species contributing primarily Lipid 567. It should be emphasized that the preferential accumulation of complex lipids containing 3-OH *iso* C_17:0_ in subgingival plaque samples at periodontitis sites is associated with a proportional reduction of LPS containing this fatty acid in the same samples [1]. Further research is needed to determine the underlying reason for this inverse relationship where bacterial lipids containing 3-OH *iso* C_17:0_, including the serine/glycine lipids and the phosphorylated dihydroceramides, are preferentially accumulating in subgingival plaque at periodontitis sites relative to LPS from the same organisms.

A recent report shows that both Lipid 567 and Lipid 1256 are TLR2 ligands but Lipid 1256 is considerably more potent than other serine/glycine lipids in engaging TLR2 [4]. This would suggest that teeth, subgingival calculus and subgingival plaque containing a higher percentage of Lipid 1256 would likely possess a greater pathogenic potential to the host. Lipid 1256 isolated from *P. gingivalis* was recently reported to significantly promote monocyte release of TNF-α and IL-1β from freshly isolated human peripheral blood monocytes [4]. This lipid was also shown to engage human TLR2/TLR6 in transfected human embryonic kidney cells [4]. Bone loss in experimental animals orally gavaged with *P. gingivalis* is reported to be mediated through engagement of TLR2 [7–9]. By contrast, the LPS receptor TLR4 was not required to mediate bone loss in mice orally gavaged with *P. gingivalis* indicating that LPS is not likely a primary innate immune ligand involved with *P. gingivalis*-mediated bone loss in mice. Of the factors produced by *P. gingivalis* that are established to engage TLR2, serine/glycine lipids specifically engage TLR2/TLR6, not TLR2/TLR1, and Lipid 1256 is the most potent serine/glycine lipid in this regard [4]. In addition, a recent report identified a lipoprotein lipase sensitive contaminant of *P. gingivalis* LPS preparations that specifically engaged TLR2/TLR1 [27]. *Tannerella forsythia* has been shown to engage TLR2/TLR1 through a leucine-rich repeat family, BspA [28]. In contrast, we previously reported that Lipid 1256 preferentially engages TLR2/TLR6 over TLR2/TLR1 [4]. Lipid 1256 therefore engages a different Toll-like heteroreceptor complex than the other TLR2 ligands reported for *P. gingivalis* and *T. forsythia*. The role of TLR6 vs. TLR1 in mediating bone loss by *P. gingivalis* and *T. forsythsus* will be the subject of future research. Though other members of the Bacteroidetes phylum produce Lipid 1256, these bacteria are not necessarily elevated at periodontitis sites as *P. gingivalis* and *T. forsythia* are [19]. Exposure of gingival tissues to these organisms and subgingival calculus likely increases delivery of Lipid 1256 to the gingival tissues thereby promoting inflammatory cell release of cytokines important in tissue destructive events such as bone loss. We are currently evaluating the potential of Lipid 1256 to directly promote osteoclast formation and activation.

The findings described in this report document the disease relevance of a newly described class of lipid mediators. The evidence provided here indicates that serine/glycine lipids, particularly Lipid 1256, are distributed throughout periodontally-diseased dental tissues and are recovered in critical bacteria associated with the expression of periodontitis. This report therefore provides a novel paradigm for exploring the manner in which these lipids can be delivered to tissues so as to locally increase chronic inflammation and local tissue destruction including alveolar bone loss. Localized (site specific) tissue destruction is a well-established feature of periodontitis and the correlation between subgingival calculus and increased attachment loss on a site specific basis has been reported [29]. Calculus is reported to be more prevalent on interproximal surfaces and least prevalent on buccal surfaces, following the pattern of attachment loss [30]. Bacterial lipids are elevated at sites where subgingival calculus accumulates and along with the coexistent subgingival plaque, may drive inflammation and localized tissue destruction. In summary, this report establishes the presence of specific serine/glycine lipids on periodontally-diseased teeth and in gingival tissues from periodontitis sites, and suggests that serine/glycine lipids are contributing to the progression of periodontitis. Further work is necessary to clarify the precise effects of these serine/glycine lipids on the wide range of cells known to be important in the tissue destructive processes associated with periodontitis.

## Data Availability Statement

The original contributions presented in the study are included in the article/Supplementary Material, further inquiries can be directed to the corresponding author/s.

## Ethics Statement

The studies involving human participants were reviewed and approved by Institutional Review Board, 263 Farmington Avenue Farmington. Written informed consent for participation was not required for this study in accordance with the national legislation and the institutional requirements.

## Author Contributions

FN, KB, Y-HW, and EC were involved with design of the clinical portion of the project and gathering clinical samples. FN, RC, QZ, MD, and FD contributed to the intellectual development of the project. MD provided bacterial samples for analysis. AP was responsible for the mass spectrometric analysis of lipid samples. All authors were critical reviewers of the manuscript.

## Conflict of Interest

The authors declare that the research was conducted in the absence of any commercial or financial relationships that could be construed as a potential conflict of interest.
